# Glucose Toxic Effects on Granulation Tissue Productive Cells: The Diabetics' Impaired Healing

**DOI:** 10.1155/2013/256043

**Published:** 2012-12-26

**Authors:** Jorge Berlanga-Acosta, Gregory S. Schultz, Ernesto López-Mola, Gerardo Guillen-Nieto, Marianela García-Siverio, Luis Herrera-Martínez

**Affiliations:** ^1^Tissue Repair and Cytoprotection Research Group, Center for Genetic Engineering and Biotechnology, Playa, CP 10600 Havana, Cuba; ^2^Institute for Wound Research, University of Florida, Gainesville, FL, USA; ^3^Biomedical Research Direction, Center for Genetic Engineering and Biotechnology, Playa, CP 10600 Havana, Cuba; ^4^General Direction, Center for Genetic Engineering and Biotechnology, Playa, CP 10600 Havana, Cuba

## Abstract

Type 2 diabetes mellitus is a metabolic noncommunicable disease with an expanding pandemic magnitude. Diabetes predisposes to lower extremities ulceration and impairs the healing process leading to wound chronification. Diabetes also dismantles innate immunity favoring wound infection. Amputation is therefore acknowledged as one of the disease's complications. Hyperglycemia is the proximal detonator of systemic and local toxic effectors including proinflammation, acute-phase proteins elevation, and spillover of reactive oxygen and nitrogen species. Insulin axis deficiency weakens wounds' anabolism and predisposes to inflammation. The systemic accumulation of advanced glycation end-products irreversibly impairs the entire physiology from cells-to-organs. These factors in concert hamper fibroblasts and endothelial cells proliferation, migration, homing, secretion, and organization of a productive granulation tissue. Diabetic wound bed may turn chronically inflammed, procatabolic, and an additional source of circulating pro-inflammatory cytokines, establishing a self-perpetuating loop. Diabetic fibroblasts and endothelial cells may bear mitochondrial damages becoming prone to apoptosis, which impairs granulation tissue cellularity and perfusion. Endothelial progenitor cells recruitment and tubulogenesis are also impaired. Failure of wound reepithelialization remains a clinical challenge while it appears to be biologically multifactorial. Ulcer prevention by primary care surveillance, education, and attention programs is of outmost importance to reduce worldwide amputation figures.

## 1. Introduction

What represents today a worldwide pandemic of a noncommunicable disease, diabetes mellitus, has two principal clinical forms identified as types 1 and 2. The former is a condition in which by autoimmune mechanisms pancreatic *β*-cells are eventually destroyed with an absolute insulin deficiency [1]. Type 2 diabetes mellitus (T2DM) is the most prevalent form of the disease and recently acknowledged not as a single clinical condition, but importantly, as a group of metabolic disorders. Diabetes, in general, causes chronic hyperglycemia and a wide range of downstream metabolic disturbances and multiorgan complications [2]. It is notorious, however, that although insulin secretion collapse, peripheral insulin resistance, and/or receptors' activity failure play a definitive role for the onset of sustained hyperglycemia in T2DM, a large portion of body glucose is cleared by insulin-independent mechanisms, derived from the ability of plasma glucose to influence its own clearance by a mass action effect [3]. T2DM usually most common in adult subjects exhibits a slow, silent, and insidious evolution. Hyperglycemia and its adjoined biochemical consequences undermine the whole tissues being sufficient to orchestrate irreversible systemic complications, from which the cells comprised in soft peripheral tissues and vascular structures do not escape. Lower extremities ulcerations and the potential for amputation are currently acknowledged as members of the list of diabetes complications [4].

Surgeon Davies Pryce put forward as early as 1887 the link between diabetes and foot ulceration by writing in The Lancet that “diabetes itself may play an active part in the causation of perforating ulcers” [5]. However, despite the years of efforts and research, the pathogenesis of impaired wound healing in diabetes remains incompletely elucidated [6]. This poor-healing condition appears to be a multifactorial process, which includes the amalgamation of systemic and local factors that ensure a perpetual forward loop up to chronification. Along this path, the cells seem to progressively wipe out their ability to trigger evolutionarily imprinted mechanisms as migration, proliferation, and transdifferentiation becoming increasingly statics. Thus, diabetic wounds do not only become chronic by a concept of aberrant healing trajectory within a physiological time frame, but also by the asynchrony on the sequence of overlapping events that make up the tissue repair megaprocess. Broadly speaking, diabetes impairs most if not all these events. Thus, the challenge that represents the diabetic wound healing failure is the clinical gross expression of an outstanding array of biochemical and cellular disorders [7]. These ideas are supported in the clinical arena by the alarming statistics of amputations around the world every year [8].

The healing process in diabetes is also jeopardized by the patient's susceptibility to infection due to deficiencies on the innate immunity. Although the diabetic wound bed may be adversely overwhelmed by inflammatory cells, it does not represent an overt antibacterial protection. By the contrary, the diversion of glucose to the polyol pathway affects bacterial killing by reducing neutrophil opsonophagocytosis. Furthermore, hyperglycemia-induced reactive oxygen species (ROS) deregulated the innate immunity via an overactivation of NF-kappaB (NF-*κ*B), thus amplifying the absurd inflammation and intoxicating the wound milieu [9, 10]. Peripheral arterial disease, leading to ischemia or lower limb hypoperfusion is associated with the most severe outcomes, including lower probability of healing, longer healing times, higher probability of ulcer recurrence, greater risk of amputations, and potentially higher mortality [4]. Cells harvested and cultured from hypoperfused granulation tissues orchestrate a molecular program of arrest and senescence (Jorge Berlanga-Acosta. Manuscript accepted. Int Wound Journal). The outcome of the combination “healing failure” and “infection susceptibility” untowardly contributes to amputation. Here we review the current lines of evidences on the toxic resonance of acute and long-term exposure to high glucose on the two main cells for the granulation tissue organization: fibroblasts and endothelial cells. We have included a characterization of the diabetic granulation tissue organizational disorders and the challenge that represents its ultimate process, wound reepithelialization. The literature search was based on English language articles downloaded from Pubmed and Bioline International (http://www.bioline.org.br/) data sources.

## 2. Consequences of Glucose Overload Toxicity on Fibroblasts and Endothelial Cells

### 2.1. Fibroblasts

The fibroblast is central to the wound healing process by secreting, contracting, and remodeling the extracellular matrix (ECM). They also secrete growth factors as important messengers for mesenchymal-to-mesenchymal and epithelial-mesenchymal communication, especially for establishing the emerging basement membrane and subsequent reepithelialization. Therefore, any impediment to fibroblast function is deterrent for normal wound healing and may result in chronic, nonhealing wounds. The fibroblast, when engaged in fibrogenesis, displays the highly activated phenotype characteristic of myofibroblasts. Although their origin has not yet been definitely elucidated; proliferation of preexisting adjacent dermal fibroblasts and, probably, recruited from the bone marrow has been documented [11]. Under the high glucose burden imposed by diabetes, cutaneous and extra cutaneous fibroblasts appear perturbed; and for many years, *in vitro* models recreating “clinical hyperglycemia” have proved to disrupt normal fibroblasts physiology and derange the secretion of extracellular matrix ingredients. These experiments have suggested that high glucose concentration is the proximal detonator of a downstream cascade of molecular disturbances for the skin fibroblast [12].

Rowe and coworkers pioneered the *in vitro* models that demonstrated that in diabetics' cutaneous fibroblasts; synthetic, proliferative, and secreting capabilities are reduced [13]. Other parallel studies, in which high glucose concentrations were introduced, proved to inhibit fibroblast proliferation, while the cells turned resistant to proliferate to growth factors such as insulin-like growth factor type-I (IGF-I) and epidermal growth factor (EGF) [14]. Following this attractive targets, Goldstein's findings allowed for establishing the hypothesis that diabetics fibroblasts replicative life span did proportionally decline with diabetics' predisposition under normal glucose concentrations, concluding that a persistent, heritable abnormality is present in mesenchymal tissues of overt diabetics and genetically predisposed subjects [15]. Years later, Goldstein also announced that cells obtained from insulin-dependent or insulin-independent diabetics not only exhibit abnormal replicative capacity *in vitro*, but that the aging process appeared more precociously than in nondiabetic counterparts [16]. Other studies showed that the addition of conditioned media from non-insulin-dependent diabetes mellitus wound fibroblasts induced a dose-dependent inhibition in normal fibroblast proliferation which appeared related to elevated L-lactate levels [17]. This replicative refractoriness of diabetic fibroblasts has been reproduced by different groups in subsequent years [18], thus confirming the need for additional external supplements to ensure cell cycle progression [19]. Accordingly, Loots and coworkers demonstrated the need of the simultaneous rather than the sequential addition of different growth factors combinations for diabetic ulcer fibroblasts in order to induce a proliferative response [20]. In addition to the onset of a quiescent and senescent phenotype of diabetic wound fibroblasts, their ability for horizontal and vertical migration is also dramatically impaired when compared to normal donor cells in different migration assay as in the modified Boyden chamber haptotaxis assay [21]. Most of these attributes are reproduced under acute exposures to high glucose concentrations so that migration speed is reduced by ~40% associated to a decrease in cell directionality and to nonproductive protrusive events, as loss of cell polarization, consistent with the increased activity of Rac1 and the projection of multiple lamellipodia. This experiment concluded that the generation of reactive oxygen species (ROS) may lie behind these abnormalities as they were partially or completely rescued by treatment with N-Acetyl-Cysteine (NAC) [2]. In contrast to the cellular reactions when exposed to high glucose *in vitro*, full-thickness wounds induced in nondiabetic pigs exposed to a local hyperglycemic environment exhibited no difference in wound closure when compared with normoglycemic controls, suggesting that delayed wound healing by diabetes is a far more complex phenomenon than circumscribed high-glucose concentration itself [22]. As a consequence of the cutaneous accumulation of advanced glycation-end products (AGEs), the skin increases its chronological age. One of the AGEs precursors is 3-deoxyglucosone (3DG). Fibroblasts cultured on 3DG-treated collagen reduce the ability to migrate efficiently since 3DG increases its adherence to the matrix. Additionally, the authors describe a higher level of misfolded proteins [23]. Using the same experimental system, this group demonstrated two years later that the inhibition in fibroblast migration, proliferation, and collagen expression by exposure to 3DG-collagen was mediated via extracellular regulated kinase 1/2 (ERK1/2) and Akt downregulation through activation of p38 MAPK (Mitogen-Activated Protein Kinase), indicating that p38 is a key signaling molecule that plays an opposite role during times of cellular growth and cellular stress [24]. Enriching the above findings, this group also demonstrated that 3DG-modified collagen induces oxidative stress, endoplasmic reticulum stress, and apoptosis via caspase-3 activation.

Oxidative stress appeared dependent on the upregulation of NAD(P)H oxidase 4 (Nox4), a reactive oxygen species Nox homologue, which appeared activated by p38 MAPK. Proximal to this cascade is the effect caused by the interaction of the modified collagen with 3DG, which signals to the fibroblast by interacting with integrins alpha-1/beta-1 (*α*1*β*1) and not through the canonical receptor for advanced glycation end-products (RAGE) [25]. Other groups have also demonstrated the induction of cutaneous fibroblasts apoptosis through cytoplasmic and mitochondrial pathways by plating the cells in an AGE-enriched environment made up by Ne-(carboxymethyl)lysine (CML)-collagen, which primarily activated the classic AGE receptor (RAGE) [26]. A subsequent study elegantly demonstrated that after AGEs-RAGE interaction, oxygen species generation is increased, activating both NOS and ceramides, which in turn activates p38 and c-Jun N-terminal protein kinase (JNK). Activated p38 and JNK triggers a cascade that leads to amplify caspase-3 activity, whereas activation of Forkhead box O class 1 (FOXO1) increases the likelihood of apoptosis through enhanced expression of proapoptotic genes [27]. Under a number of circumstances, FOXO transcription factors induce BIM and other proapoptotic genes expression.

In addition to the deleterious effects of glucose and its derivatives, diabetic fibroblasts exhibit particular features. Literature documents that diabetic mice fibroblasts show a severe impairment in VEGF production under normoxic and hypoxic conditions in addition to an increased pro-degradative activity due to the high expression of matrix metalloprotease type 9 (MMP-9) [28]. Similarly, diabetic pigs exhibit an impaired healing that is accompanied by a reduction of IGF-1 in the wound milieu [22]. Studies with human fibroblasts have confirmed the prodegradative phenotype by the increased MMP-2 and MMP-3 production and reduced collagens gene expression [29]. Human diabetic fibroblasts also exhibit a failure in nitric oxide (NO) production, which is concomitant to elevations in MMP-8 and -9 [30]. The fact that these fibroblasts fail in secreting NO is particularly negative given its role for wound healing. Conversely, NO donors' administration has shown to stimulate cell proliferation and restore the balance of MMPs [31].

**Figure 1:**
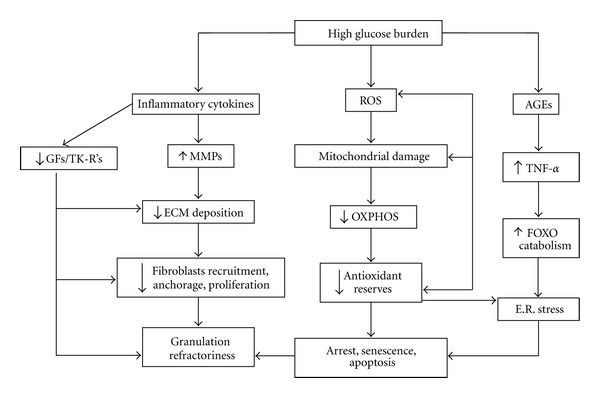
Negative impact of high glucose levels on cutaneous fibroblasts biology. Short- or long-term exposure to high glucose concentrations is toxic for cutaneous fibroblasts suppressing the cells' biological activities. The fibroblasts become reactive but not active. The high glucose burden engenders and uncontrolled production of ROS within the mitochondria with three major consequences: detriment on the OXPHOS reactions, depletion of the cells antioxidant reserves, and amplification of the mitochondrial dysfunction due to ROS-mediated attack to its DNA. Under this scenario apoptosis may prevail. ROS also may lead to cell cycle arrest due to p53 and p21 upregulation and nuclear compartmentalization. Alternatively, high glucose concentrations may impose a proinflammatory program within the wound by perpetuating a special population of macrophages (M1) so that fibroblasts become intoxicated and suppress the secretion of ECM ingredients. Conversely, the inflammation mediators fuel the secretion of MMPs. The negative balance of ECM inhibits fibroblasts chemotaxis, homing, anchoring, and proliferation. The proinflammatory environment inhibits the secretion of numerous growth factors with fibro angiogenic potential as TGF-*β*, PDF, EGF, and so forth and interferes with the signaling pathways of the TK-R's. Inhibition of the TK-R's downstream networking entails the suppression of positive forces for a balanced control of granulation tissue repopulation with productive cells. The accumulation of AGEs activates the AGE/RAGE axis, which further amplifies local inflammation and reactivity by increasing the secretion of TNF-alpha and adhesion molecules. This cytokine interferes with insulin and growth factors signaling, TGF-*β*1 for instance, which further amplifies the obstruction of the PI3K/Akt/mTOR/Cyclin D axis. The balance against this vital axis promotes the nuclear compartmentalization of representative of the FOXO family members, which contributes to catabolism, senescence, arrest, and apoptosis. In this prooxidative environment, it is common to activate cells autophagy in response to the accumulation of missfolded proteins. Thus, all these factors converge to slow down granulation tissue outgrowth.

It seems that amplification of oxidative stress acts as a primary culprit in harming fibroblasts biology in diabetes, involving electron transport in mitochondria. High intracellular glucose levels increase the electron transport chain in mitochondria during oxidative respiration, leading to formation of O2- and the generation of various reactive oxygen species derivatives in the mitochondria. Other sources of oxidative stress in diabetes include glucose autooxidation, the polyol pathway with ensued depletion of antioxidant reserves and the formation of AGEs [32]. Chronic hyperglycemia-induced mitochondrial ROS stimulate various signaling pathways that amplify inflammation and cell death. They include protein kinase C (PKC), JNK, and p38/MAPK [31]. According to an excellent review by Ponugoti et al. [33]; ROS leads to the activation of members of the FOXO family. This is a family of transcription factors with apparently opposing roles that may defend cells against oxidative stress but also promote cell-cycle arrest in G1 by inducing p27kip1 [34]. FOXO1 activation appears elevated in diabetic connective tissue cells and mediates AGEs and tumor necrosis factor-alpha- (TNF-*α*-) induced apoptosis both of which are abundant in diabetic connective tissue [35]. FOXO1 limits wound healing by inhibiting fibroblasts proliferation and enhancing their apoptosis [35, 36]. Interestingly, insulin inactivates FOXO1 via Akt leading to its nuclear export and degradation. Defective insulin action in the skin has been proposed as an important mechanism contributing to wound healing defects in diabetes. Perhaps the assorted constellation of the hormone's pharmacological bounties (increased expression of endothelial nitric oxide synthase, vascular endothelial growth factor, and stromal-derived factor-1*α*/SDF-1*α*) observed in experimental and clinical wounds when insulin is topically administered may be attributable to FOXO1 neutralization. Curiously, the acceleration of wound healing occurs in parallel to a local recovery in the expression of proteins involved in insulin signaling pathways [37]. Aside from the above arguments, these preclinical and clinical findings are not surprising in light of the potent anti-inflammatory, proanabolic, and cytoprotective actions of insulin [38], which extend beyond the exclusive regulation of glucose homeostasis [3].  Figure 1 depicts the main pathogenic players and their interconnection as an attempt to summarize the high glucose-triggered fibroblasts damage process.

Despite the prolific investigation conducted during all these years, still questions remain to be answered in relation to *ex vivo* diabetics' fibroblasts behavior.

1. Why diabetics' fibroblasts evoke behavioral traits in culture mirroring the donor's tissue, even when grown under optimized oxygenation, nutrient, growth factors, and glucose supply?
2. Is there any sort of “behavioral imprinting” so that they are reminiscent from a diabetic donor?
3. Why cultured fibroblasts from both ischemic and neuropathic ulcers exhibit different ultrastructural morphology and organize the monolayer in a privative manner?
4. Is there any epiphenomenon beyond the irreversible glycation sustaining the “impersonation” of the *in vivo* traits?

### 2.2. Endothelial Cells

Angiogenesis is a comprehensive term that indicates the physiological process involving the growth of new blood vessels or neovascularization. This is a vital process for embryological growth, tissue development, and wound healing. Different growth factors families as vascular endothelial growth factors (VEGF), fibroblast growth factor (FGF), angiopoietins, platelet-derived growth factor (PDGF), transforming growth factor-*β* (TGF-*β*), in collaboration with other proteins as integrins, cadherins, and ephrins, regulate angiogenesis by promoting endothelial cells recruitment, proliferation, migration, coopting, and collar stabilization. There is an enormous and ever-growing body of evidence indicating the close correlation between hyperglycemia and the abnormalities in endothelial function and morphology [39]. The UK Prospective Diabetes Study (UKPDS) and Diabetes Control of Complications Trial (DCCT) found microvascular disease and hyperglycaemia to be intrinsically related. Thus, anomalous angiogenesis is a hallmark of both type forms of diabetes, which is clearly and early observable during the process of granulation tissue growth, a condition that has been successfully reproduced in animal models [40]. For subjects with macrovascular disease, the defective angiogenesis prolongs and disturbs the healing process. The concept of abnormal angiogenesis extends beyond the wound, given the inability of these patients to create collateral circuits due to VEGF-dependent monocytes dysfunction [41]. Furthermore, insulin has a dramatic impact on the endothelial homeostasis by its ability to stimulate NO release via a cascade that involves activation of the phosphatidylinositol 3-kinase (PI3K)-Akt signaling and endothelial nitric oxide synthase (e-NOS) phosphorylation. The later being of paramount importance in angiogenesis and wound healing as described below [42].

As depicted for fibroblasts, high glucose and the glycated by-products exert a toxic effect on endothelial cells and the vascular wall in general. In parallel, the endothelial cells *per se* seem to be a very sensitive target to high glucose. Endothelial dysfunction is intricately related to insulin resistance through the stimulatory effects of insulin on glucose disposal and NO production in the endothelium. Today, vascular dysfunction remains as a major cause of morbidity, amputation/disability, and mortality in diabetic patients. Even after achieving the successful reperfusion of an ulcerated lower extremity, the healing process is slow and torpid. Therapeutic angiogenesis has been pursued for years, but the clinical results have shown relatively limited outcomes [43–45]. High glucose concentrations have been associated with endothelial metabolic dysfunction *in vitro* and *in vivo* and as for multiple physiological processes; insulin and its downstream signaling regulate most of the endothelial cell functions [46]. High glucose ambient has been shown to disturb endothelial cells cycle, increase DNA damage, and delay endothelial cells replication, as inducing excessive cell death [47]. In addition, high glucose also prevents NO-induced inhibition of vascular smooth muscle cells (VSMC) migration [27] thus contributing to Monckeberg's media thickening. *In vitro* models simulating “normoglycemia” and “hyperglycemia” have demonstrated that under high glucose ambient, proliferation and tube formation of dermal microvascular endothelial cells appear impaired [48]. Furthermore, high glucose levels selectively trigger apoptosis in cultured endothelial cells as has been demonstrated by different laboratories [49]. High glucose induces the upregulation of TNF-*α* level concomitant to the death receptors TNF-R1 and Fas in a variety of cultured endothelial cells [50]. Under this ambient, Bax protein expression increases, cytochrome c is released, subsequently conjugating to Apaf-1 and triggering a caspase cascade-induced death [51].

Hyperglycemia-induced oxidative stress promotes inflammation through increased endothelial cells damage, microvascular permeability, and uncontrolled release of proinflammatory cytokines, including TNF-*α*, interleukin-1*β* (IL-1*β*), and interleukin-6 (IL-6), ultimately leading to decreased insulin sensitivity and diabetic vascular complications. Moreover, hyperglycemia-induced FOXO also plays an important role in the induction and amplification of proinflammatory cytokines production. FOXO1 directly binds to IL-1*β* promoter and increases its expression in macrophages [52].

Hyperglycemia and the accumulation of AGEs disturb the role of angiogenic growth factors as VEGF, and its receptor, its signaling pathway, thus disrupting endothelial proliferation, migration, and endothelial progenitor cells (EPCs) recruitment and release from bone marrow [53]. Insulin resistance disrupts the NO-mediated angiogenic positive regulation over angiogenic growth factors such as VEGF, FGF, and TGF-*β* [53]. Studies using streptozotocin-induced diabetic mice with simultaneous hind-limb ischemia have suggested that the angiogenic responses remain preserved even under the diabetic state, and that 40 to 50% reduction of platelet-derived growth factor-BB (PDGF-BB) expression is responsible for the induction of functional and morphological vascular abnormalities and pericytes apoptosis. Conversely, PDGF-BB external supplementation was sufficient to prevent limb autoamputation, an event also reproduced with a PKC inhibitor that restored the expression of endogenous PDGF-BB [54].

**Figure 2:**
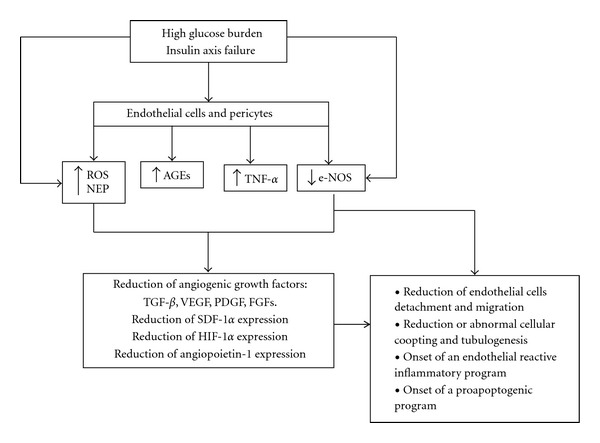
Negative impact of high glucose levels and failure of the insulin system on vascular cells. Endothelial cells are a sensitive target for high glucose concentration and especially for the insulin receptor downstream signaling attenuation. Similarly, pericytes, which are key cells for the angiogenic process to succeed, are targeted by these factors. The endothelial cells metabolic response to the above proximal triggers engenders the accumulation of superoxide and hydroxyl reactive groups. These prooxidative elements disrupt the physiological pathways for NO metabolism, accumulating toxic nitrosylation end-products. In this context, AGEs are precipitately formed and accumulated within the vascular wall. Different pathways converge to induce TNF-*α* overproduction. The proximal triggers and all these factors also contribute to disrupt e-NOS activity having as net result a deficit in endothelial NO, the inability for vasodilation, and the suppression of endothelial cells proliferative reserves. This circle is further amplified due to the concomitant reduction in the pool of critical angiogenic factors involved in the regulation of vascular regeneration. Eventually endothelial cells and pericytes may onset a pro-apoptogenic program, which will hinder granulation tissue perfusion and wound healing.

The glycation of collagen and other proteins within the wound extracellular matrix and AGEs accumulation bring catastrophic consequences for the angiogenic reaction with inhibition of angiogenesis *in vivo*. The fact that angiogenesis is restored by aminoguanidine treatment reinforces the antiangiogenic role of AGEs [55]. Angiogenesis is a multifaceted process demanding an appropriate, nonglycated extracellular substrate. This is clearly illustrated by the fact that PDGF-BB anchors to different components of the ECM under physiological conditions acting as a natural depot and slow release system for the growth factor. Local PDGF unavailability has proved to impair the coverage of newly formed vessels with mural cells and local pericytes [56]. This evidence reinforces the pathophysiological impact of high glucose toxicity, the release of proinflammatory cytokines, and the activation of the intrinsic mitochondrial-mediated apoptotic signaling pathway on endothelial cells. In summary, endothelial cells exposed to excess glucose trigger the onset of a pro-inflammatory profile turning these cells into a cytokines and ROS manufacturing plant. The agonistic stimulation of the AGEs receptor is able to mount the same response leading to apoptosis and vascular ruin. The pathogenic effects of hyperglucose associated to insulin axis failure on endothelial cells are summarized in Figure 2.

Compelling evidence indicates that at least a portion of the hyperglycemia-mediated endothelial damages and dysfunctions are associated with an impaired mitochondrial activity resulting in mutations of mitochondrial DNA, due to a disproportionate reactive oxygen radicals production, leading to an inflammatory reaction and apoptosis [57]. As a matter of fact mitochondrial DNA has a much higher mutation rate than nuclear DNA because it lacks histones and is exposed to the direct action of oxygen radicals while its repair system is limited. Therefore, ROS appear to play a pivotal role in systemic endothelial deterioration and biological aging [58]. As described, ROS generation enhances FOXO1 activation and induction of several classes of genes that regulate endothelial cell behavior, including pro-inflammatory factors and eventually the execution of apoptosis of endothelial and adjacent cells [33]. ROS-mediated lipid peroxidation appears to impair most healing events, contributing to growth factors reduction, keratinocytes migration failure, slow or torpid fibroplasia, delayed contraction, and matrix remodeling, not to mention abnormal angiogenesis [59]. Under experimental conditions, the pharmacological intervention with a chemical inhibitor of lipid peroxidation proved to reduce the local edema and to stimulate reepithelialization, neovascularization, proliferation of fibroblasts, and synthesis and maturation of the extracellular matrix. A parallel finding was the normalization of VEGF mRNA expression and secretion in those diabetic mice. This further supports the view that lipid peroxidation perturbs VEGF production [60]. An extraordinary background has accumulated about the role of NO in vascular biology in diverse horizons as ischemia, inflammation, and neovascularization. Impaired endothelium-dependent NO-mediated relaxation occurs in both cellular and *in vivo* models [61]. Many of the metabolic conditions associated with diabetes are conditioned by failure in NO synthesis or its degradation. In this respect, the integrity of the Akt/e-NOS coupling pathway for a normal endothelial function appears compulsory [62]. Hyperglycemia is also associated to a deficit in tetrahydrobiopterin (BH4) and to an increase in arginase expression, which attempt against NO synthesis and normal endothelial functions such as vascular remodeling responses [63]. The increased generation of peroxynitrite levels under high glucose conditions contributed to deplete cellular antioxidant reserves as to activate NF-*κ*B and consequently the expression of the inducible form of nitric oxide synthase (iNOS), intercellular adhesion molecule-1 (ICAM-1), and other inflammatory mediators [64].

Endothelial Progenitor Cells (EPCs) are active players for the maintenance and repair of endothelial cells. They participate in angiogenesis as they proliferate, migrate, and differentiate and are a source for proangiogenic factors and cytokines [65]. Multiple lines of evidence indicate that the number of circulating EPCs is decreased under both clinical forms of diabetes, which is likely to be involved in the pathogenesis of vascular complications [66]. Under experimental diabetic conditions, the EPCs number appears significantly, decreased in the bone marrow as in the peripheral blood, which was reverted by treating the mice with insulin [67]. In general, the bone-marrow-derived EPCs in the diabetic patients are considered as dysfunctional, producing fewer endothelial cells with reduced replicative and migratory potential [68]. Tamarat and coworkers have described a limited capacity of diabetic animals-derived bone marrow mononuclear cell to differentiate into endothelial progenitor cells *in vitro* as to organize tubulogenic structures when subcutaneously implanted in a matrigel plug, thus hindering the revascularization of damaged areas [66]. Over again, the activation of p38 MAPK mediated by an excessive ROS generation has been aimed as responsible for the EPCs impaired proangiogenic potential *in vivo* by limiting cells proliferation and differentiation [69]. As to fully divert the physiological role of EPCs in tissue repair and angiogenesis, the duet hyperglycemia-ROS stimulate the EPCs to produce pro-inflammatory cytokines and to shift NO production by elevating i-NOS and decreasing e-NOS [70]. As described for other cells, AGE treatment disrupts EPCs physiology thus leading to a downregulation of e-NOS and Bcl-2 expression, as well as an elevation in cyclooxygenase-2, Bax, NF-*κ*B, and caspase-3 in a MAPK- (ERK/P38/JNK-) dependent manner [69].

The diabetes-mediated vascular damage is perhaps the most outspoken and ancestrally identified emblem of diabetes. It is varied and broad as it is the concept of systemic endothelial dysfunction. Diabetes distorts the angiogenic program to ironically culminate with a maldistribution of soluble angiogenic factors: shortage where and when required (lower extremities skin) but overproduced where and when not needed (retina). It is also challenging, to understand *how *and *why* microvascular morphological changes that recreate chronic, life-time processes are readily identified in a 7-day-old granulation tissue fragment, even in compensated patients. This incites to investigate which are the diabetes' operational local and/or systemic forces that can disrupt vascular morphogenesis.

## 3. Failure of Granulation Tissue Onset and Progression

Once we have described the main consequences of high glucose/hyperglycemia on the two principal architects of the granulation tissue: fibroblasts and endothelial cells, we are intended to recapitulate the most distinguishing features on the onset of the granulation process in diabetic cutaneous wound healing.

Tissues' regenerative capabilities have been neglected along the species evolution; thus, scarring process has emerged as an urgent alternative to favor the structural and functional restoration of a wounded zone. Within these events, the process of granulation tissue formation is pivotal as it constitutes a sort of living, temporary aggregate of cells and proteins, acting as a welding material until the tissue's continuity is restored. However, the reluctance to trigger and sustain the out-growth of a productive granulation tissue with an appropriate extracellular matrix is typical in diabetic patients, and particularly if ischemia concurs. As mentioned, these wounds are characterized by a proliferative arrest, proinflammed, prooxidant, and prodegradative phenotype [71].

This stubbornness and slowness to heal in diabetes is conditioned by systemic and local factors that in complicity counteract intrinsic reparative mechanisms. In a broad systemic context, inflammation and the anabolic deficit can be conceptually mentioned. Diabetic patients with foot ulceration bear a specific and nonrandom alteration of the immune status with an active upregulation of circulating levels of acute-phase proteins, cytokines, and chemokines that impose a chronic systemic inflammatory profile and amplify local wound inflammatory networks [72]. The systemically elevated levels of pro-inflammatory response markers and the wound's expression of cytokines and chemokines are among the culprits of the abnormal repair mechanism [73]. Another factor to be considered is that diabetes *per se* is a metabolic disease in which fuels metabolism is perturbed given the rupture of one of the most important anabolic axis of the organism: insulin/insulin-like growth factor type-I. The role of insulin in wound healing is well known by its anabolic effect on wound protein balance favoring synthesis and preventing degradation [74, 75]. IGF-1 has a similar effect on stimulating wound tissue anabolism. Both insulin and IGF-1 appear to act in part by the induction of ATF4 (CREB2), essential for the activation of mammalian target of rapamycin complex 1 (m-TORC1), which in turn is required for protein synthesis via FOXO-dependent genes repression [76]. We do not rule out that the diabetes-concomitant deficit of incretins could participate in the negative anabolic balance observed in such wounds. Glucagon-like peptide-1 (GLP-1) in addition to its antihyperglycemic actions is endowed with a vast number of multi-organ cytoprotective, trophic and antiinflammatory effects [77]. In support to the GLP-1 action is the study by Ta and coworkers with alogliptin, a specific inhibitor of dipeptidyl peptidase-4 (DPP-4) which showed to inhibit macrophage-mediated inflammation response and to speculatively promote tissue remodeling by inhibiting the expression of different matrix metalloproteases [78].

Rapid formation and deposition of an appropriate extracellular matrix, in particular by fibroblasts, is required for an efficient cellular anchoring and homing at the wound bed. As mentioned above, the cutaneous fibroblast is a sensitive cell to high glucose, AGE-precursors, AGEs, ROS, and TNF-*α*, rapidly undergoing premature senesce, arrest or apoptosis. Fibroblasts are the main source of collagen, and the number of fibroblasts can be taken for a measure of repair by their collagen synthesis ability. It is very likely that the deficit of growth factors such as TGF-*β*1, IGF-I and PDGF could translate into a DFU with scarce extracellular matrix acumulation and impoverished cellularity. Numerous growth factors (TGF-*β*1, IGF-I, PDGF) are able to regulate the balanced expression of matrix metalloproteases and tissue inhibitors of metalloproteases (MMPs/TIMPs), while most of them exhibit an altered expression in DFU [79]. Moreover, the imbalance in the DFU milieu between TGF-*β*1 and TGF-*β*3, in which the former appears downregulated, may explain fibroblasts quiescence in terms of proliferation and secretion [80]. This phenomenon represents the deficit of one of the most potent profibrogenic and fibroblasts-mitogenic growth factors, which at the same time is able to downregulate macrophage activation [81]. The extracellular matrix represents the granulation tissue dynamic stroma that provides support for inflammatory cells, fibroblasts, and endothelial cells and allows for the chemotaxis of epithelial cells, thus hosting the reepithelialization process [82]. One of the main challenges for the diabetic wound healing is the structuring of a normal matrix in quantity and quality. In general, a poor extracellular matrix formation distinguishes DFUs, which can result from (a) diminished synthesis, (b) increased rate of degradation by proteolytic enzymes, (c) toxicity due to glycated by-products accumulation, and (d) toxicity by biofilm bacterial contaminants diffusion [83]. We deem that an important cause of the clinical dilemma of the high rate of reulcerations and ipsilateral amputations in DFU patients' shortly after reepithelialization [84] may be inherent to the qualitative composition of the scar matrix to tolerate tensile forces and mechanical stress.

**Figure 3:**
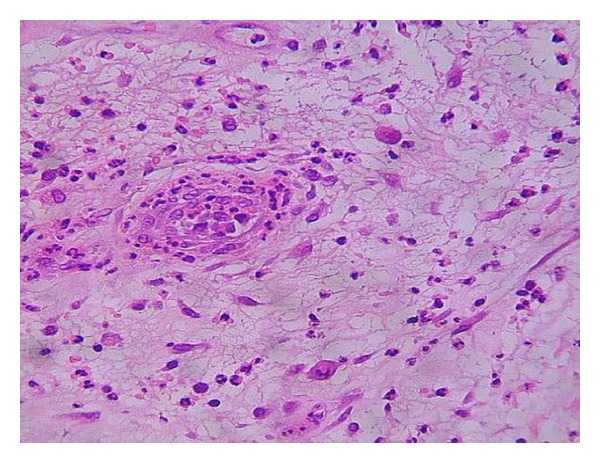
Common histological aspect of a neuropathic granulation tissue. Neuropathic granulation tissue exhibiting a scarce deposition of extracellular matrix. Note a central blood vessel with abundant surrounding heterogeneous cellularity and abundant fibrin accumulation, suggesting hyperpermeability. Hematoxylin/eosin staining, ×10 magnification.

The diabetic granulation process does not generally exhibit the orderly cascade of events that characterize normal wound healing. This has been confirmed through the histopathological analysis of granulation tissue biopsies by Loots and coworkers who described the lesions as “frozen” in a chronic low-grade inflammatory state associated to a scarce provisional extracellular matrix [85]. Our group's serial biopsies from both neuropathic and ischemic ulcers-derived granulation tissue have identified histological differences for both types of wounds in the absence of clinical infection. Polymorphonuclear cells (PMN) infiltration is intense and prolonged particularly in neuropathic wounds, co-existing with a scarce extracellular matrix accumulation in which collagen deposit is impoverished (Figure 3).

**Figure 4:**
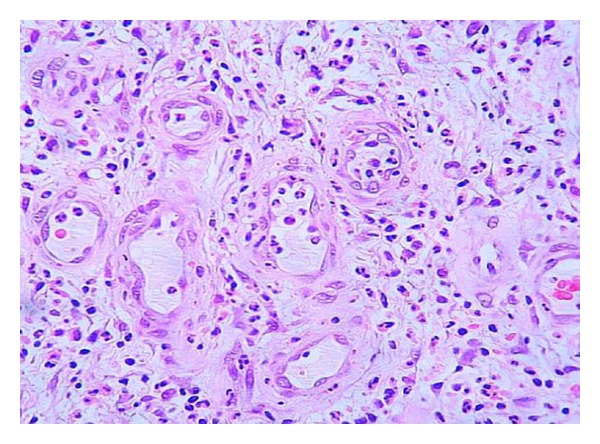
Common histological aspects of ischemic granulation tissue. Note the presence of an intense inflammatory infiltrate of round cells spread all over the tissue area. The emerging vessels appear unfunctional with thickened walls of fibrohyaline material, “hardened aspect”, and endothelial nuclei hypertrophy. Hematoxylin/eosin staining, ×10 magnification.

Under more mature stages, the neuropathics may also show an abnormal sprout of new small vessels and capillaries that may derive not from a normal angiogenic response but due to arteriovenous shunts. Our observations remind us with those of Black and coworkers who demonstrated that in neuropathic patients there exists a decrease in fibroblast proliferation and a scarce amount of collagen accumulation within the wound bed [86]. On the contrary, a broadly spread infiltration of round cells predominate in those patients suffering from wound bed ischemia, associated to a fibrohyaline matrix of “hardened” aspect and abnormal angiogenesis in which vascular wall cellular mosaicism, precocious media thickening, endothelial nuclei hypertrophy and many other defects can be identified (Figure 4). It is likely that the combination of arterial hypoperfusion and glucose toxic derivatives imprints a particular pattern of damage to the morphogenesis of vessels in the wound [87]. These observations incite to speculate that the biochemical microenvironment in ischemic and neuropathic diabetic wounds is different and that the inflammatory “badge” is in correspondence with the wound's most prevalent pathogenic component [88]. In contrast to acute wounds in nondiabetic subjects, the inflammatory reaction in diabetics appears prolonged [89] which sharply delays granulation tissue formation and maturation [90]. Data derived from murine diabetic models indicate that the exaggerated inflammatory reaction is related to the prolonged expression of macrophage inflammatory protein-2 (MIP-2) and macrophage chemoattractant protein-1 (MCP-1) [91]. Furthermore, the downregulation of the anti-inflammatory cytokine IL-10 in DFUs environment represents the collapse of an important inflammatory restrainer [73]. Another evidence indicates that PMNs are critical cells toward the acquisition and perpetuation of inflammation and a degradative phenotype. The granulocytes secrete TNF-*α* and IL-1*β*, which act as a triggering signal for MMPs expression via the common NF-*κ*B signaling pathway. Within the wound context, TNF-*α* stimulates its own secretion and that of IL-1*β*, which contributes to a persistent inflammatory status [92]. TNF-*α* has proved to negatively impact the repair process as it is early secreted since the inflammatory phase. Its deregulation is not only associated with persistent inflammation but also to connective tissue degradation [93]. Concomitantly, TNF-*α* mediates its antagonistic effects on TGF-*β*1 through the JNK pathway via inhibition of Smad phosphorylation, consequently reducing the expression of TGF-*β*1, and that of several downstream matrix proteins [94]. In this highly proteolytic milieu, fibronectin, collagens, growth factors, and their receptors are degraded while the wound is way down to a catabolic state [95].

Importantly, the perpetuated homing of PMN within the wound bed is associated to high local levels of elastase secretion, ROS, and reactive nitrogen species [96]. High circulating and PMNs-associated elastase levels are attributable to a poor glycemia control and are currently considered as a risk marker for the development of diabetic angiopathy [97]. Fibronectin degradation, for instance, is referred as one amongst the several causes of diabetic reepithelialization failure. Epidermal keratinocytes require of the interaction between fibronectin and its surface receptor integrin *α*5*β*1 to effectively migrate [98]. Curiously, insulin-degrading activity has also been demonstrated in the fluid of diabetic experimental and human wounds, which have been shown to correlate with the glycated hemoglobin levels [99]. The connection between NO metabolism and foot ulcer proteases profile has been described. In contrast to elevated MMP-8 and 9 displayed by the nonhealing diabetic foot wound, the concentration of NO appears significantly reduced. Diabetic skin fibroblasts treated with NO donor compounds, selectively raised NO production, increased cell proliferation, and decreased the expression of MMP-8 and -9 in a dose-dependent manner. Thus, that NO resumes the cell proliferation program and promotes the reestablishment of an antiproteases effect has emerged as argument in favor of the NO salutary effect in wound healing [30].

The link between wound cells and apoptosis was described above; we just wish to comment that in sharp contrast to wound-infiltrated inflammatory cells, which become refractory to apoptosis, granulation tissue-producing cells are sensitive to commit suicide where TNF-*α* stands as a major driving force. The negative impact of TNF-*α* level on the sensitivity of tissues to insulin has been consistently documented. Skin cells are not excluded from this effect [100]. Conclusively, any therapeutic approach aimed to neutralize TNF-*α* or to increase the wound local availability of active TGF-*β*1 would be similarly effective for stimulating granulation tissue and wound closure [101].

Chronic wounds and especially diabetic foot ulcers exhibit a highly pro-oxidant microenvironment that amplifies the cytotoxic cascade. Endothelial cells and fibroblasts, in particular senescent fibroblasts, are a prominent source for oxygen radicals, but at the same time they turn into these radicals targets which by converging mechanisms arrest cell proliferation and induce apoptosis [102]. Thus, the disturbed oxidant/antioxidant balance as the AGEs accumulation within the chronic wound microenvironment is considered a major factor, which amplifies the unrestrained and persistent inflammatory, toxic, and catabolic state of nonhealing wounds [96].

**Figure 5:**
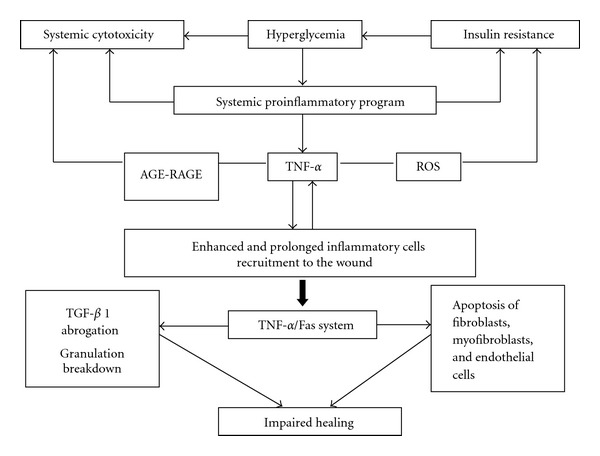
Impact of hyperglycemia on granulation tissue biology. The onset of a systemic pro-inflammatory program due to sustained hyperglycemia is associated with the elevation of circulating levels of TNF-*α*. The cytokine release is further amplified by the agonistic interaction AGE/RAGE and the generation of ROS. This preliminary triad amplifies insulin receptor resistance and a multiorgan toxicity. Excess TNF-*α* perpetuates the inflammatory infiltrate into the wound bed hindering the onset of the fibroangiogenic phase in part by abrogating TGF-*β*1 release. This TNF-*α* related growth factors deficit within the wound bed may act as a vulnerability factor for granulation tissue productive cells apoptosis.

The failure of wound contraction is a clinical hallmark of diabetic granulation tissue. Fibroblast-to-myofibroblast transdifferentiation represents a key event during wound healing and tissue repair. The contractile force, generated by myofibroblasts as a highly specialized cell, speeds the healing process of dermal wounds in healthy humans, accounting for an 80–90% of scar tissue reduction [103]. In addition, the contraction process reduces the area to be resurfaced by reepithelialization, which represents a sort of ergonomic response. In diabetic subjects, however, contraction is impaired and deep ulcers heal by the combination of granulation and reepithelialization. The classical view on dermal wound healing implies recruitment of local fibroblasts [104] followed by a subsequent process of transdifferentiation in which the fibroblasts gain a definitive phenotype of differentiated myofibroblasts by neo-expressing a-smooth muscle actin (*α*-SMA). Nevertheless, *α*-SMA expression is precisely controlled by the joint action of growth factors like TGF-*β*1 and extracellular matrix proteins like the fibronectin (FN) splice variant ED-A, as by the local mechanical microenvironment [104]. It should be noted, however, that indwelling fibroblasts in diabetic wounds are refractory to proliferate and adopt a senescent phenotype, and that TGF-*β*1, fibronectin, and other matrix proteins may appear in deficit. Hence, all these factors may contribute to the poor contractile activity. Furthermore, Goldberg and coworkers have shown that among the deleterious activities of TNF-*α* within the wound is to suppress *α*-SMA expression in human dermal fibroblasts [94].  Figure 5 integrates the cascade of deleterious factors that impact on diabetic granulation tissue onset.

If the animals-derived evidence that a high fraction of the wound myofibroblasts potentially derives from bone marrow fibrocytes is valid for humans [105]; we have already learned that diabetes negatively impacts on the general bone marrow physiology [106] and that beyond this, stromal-derived factor-1alpha (SDF-1*α*), which acts as a recruiting factor and its CXCR4 chemokine receptor are also impaired by diabetes [107]. Finally, it has been documented that the circulating acute inflammatory reactants involved in insulin resistance inhibit fibrocytes differentiation [108].

There are numerous cellular and molecular aspects unknown and that remain to be answered on the granulation tissue biology.

1. What are the molecular and cellular driving forces supporting the microscopic structural differences between neuropathic and ischemic ulcers beds?
2. What is the explanation for the “inheritance” of vascular changes as a dramatic Monckeberg media thickening in nascent arteries within an early hatching granulation tissue?
3. Why granulation tissue is histomorphologically abnormal even in metabolically compensated patients?

Reepithelialization at the clinical level, it is not a lesser important problem as most of the diabetic wounds may granulate in time, while reepithelialization is even far slower, arrhythmic, and torpid. Reepithelialization is accomplished through the combined actions of keratinocytes' dedifferentiation, proliferation, and migration requiring a complex basement membrane, emerged of the mutual interaction between mesenchymal and epithelial cells. Reepithelialization failure is therefore one of the landmarks of diabetic and other chronic wounds. The epidermal edge of a chronic wound is thick and hyperproliferative with mitotically active keratinocytes unable to migrate along the surface, and by the contrary, moving down deep into the neodermis. Therefore, it has been speculated that the nonhealing edge keratinocytes do not successfully complete either of two possible pathways: activation or differentiation. In consonance with this, one of the major issues in chronic wounds treatments is how to revert the chronic wound keratinocytes' phenotype to a proper differentiating and migratory program [109].

Glucose has shown to exhibit a direct toxic effect on keratinocytes. As for other cells grown in the presence of high glucose concentrations, human epidermal keratinocytes significantly reduce their proliferation rate and replicative life span and were rendered more susceptible to commit apoptosis [110]. Other studies also confirmed that hyperglycemic conditions abort keratinocytes' proliferative ability and their migratory response [111]. Aside from the glucose-mediated direct cytotoxic effect on the keratinocytes, AGE modification of type-I collagen and other ECM proteins impairs the integrin-mediated adhesion of keratinocytes to the basement matrix and could thus contribute to the pathogenesis of diabetic reepithelialization failure [112]. In this context, epithelial-mesenchymal interaction plays a role in establishing the profile and order of released factors regulating keratinocytes proliferation and differentiation [113].

The fact that insulin is biologically relevant for skin cells derives from the observation that insulin is an essential component for cultured human keratinocytes, demonstrating its involvement in the regulation of proliferation, survival, and metabolism [114]. Recent studies in this field document that among other roles, insulin contributes to VEGF release in skin wound cells through an A*kt*1-mediated posttranscriptional mechanism [115]. Glucose is known to affect insulin action by regulating the expression of several genes including insulin receptor at both the transcriptional and translational levels [116]. Lack of insulin receptor expression derives in reduced skin proliferation and abnormal differentiation *in vivo* [117]. Furthermore, TNF-*α* has also been implicated in epithelial cells arrest by deeply perturbing critical elements of keratinocytes' physiology, including insulin sensitivity [118].

A notorious study has provided evidence aiming at the roles of c-myc and *β*-catenin in impairing epithelial edges migration. Nuclear beta-catenin stabilization inhibits keratinocytes migration by blocking epidermal growth factor response via c-myc induction, and repressing keratins 6 and 16 expression, depleting at the end the pool of epidermal stem cells at the nonhealing edge [119]. It is therefore evident that keratinocytes migration incapability plays an important role in reepithelialization failure since cytoskeletal keratins K2, K6, and K10 have been observed diminished in DFUs [120]. Moreover, the observation that EGF response appeared blocked may have further deleterious impact. Many peptide growth factors, including members of the EGF family, accelerate wound reepithelialization *in vitro* and *in vivo* [121]. Among them, the activation of the EGF family of ligands and the receptor is of physiological significance. Furthermore, EGF receptor (EGFR) expression is transiently increased at wound margins, suggesting an active role for this receptor in wound repair. EGF stimulates both cell proliferation and motility [122], with the later being dependent on EGFR autophosphorylation and the subsequent activation of phospholipase C*γ*-1 (PLC-*γ*1). On the other hand, EGFR activation also leads to membrane ruffling and focal adhesions through activation of members of the Rho subfamily of GTP-binding proteins [123]. Recent experiments document the negative effect of the Slug null mutation as a downstream EGFR catalytic mediator for wound reepithelialization. Thus, any interference with the EGFR cascade will hamper epithelial resurfacing [124]. Classic experiments provide illustrative examples on the relevance of the epithelial-mesenchymal cross-talk and on the irreplaceable role of growth factor as a networking bridge [125] for reepithelialization. Skin-reconstitution studies have shown that bone marrow stromal cells (BMSCs), in addition to dermis-localized preadipocytes and fibroblasts distinctively promote epidermal regeneration [126]. As diabetes courses with a deficient secretion of growth factors and other chemotactic mediators in areas of tissue repair, recruitment of circulating stromal cells appears reduced; which may turn into an additional hit to that of high glucose-associated toxicity [127]. At the end, there are so many factors which may interact to obstruct chronic wounds reepithelialization that it may turn into a puzzle.

Above all, questions from the clinical practice remain and pose as a challenge for basic researchers: (1) why after wound contours surgical debridement keratinocytes migration resumes, for soon after become stunted and arrested again? (2) Why the biopsies invariably show a hypertrophic lip of epithelial cells in vertical, downward growth in spite of a horizontal polarization?

## 4. Concluding Remarks

Although diabetes *per se* is a complex disease, our contemporary understanding on the molecular mechanisms impairing wound healing in diabetes has indefectibly expanded over the last 20 years. The last few years have witnessed the birth of the notion that type 2 diabetes is not a single, unique process, whereas the concept of group of diseases has flourishing. Type 2 diabetes seems to be ethiopathogenically multifactorial and behaves as individual as the affected subject is. So is the pattern of the clinical complications, including the wound itself.

To date, all the evidence aims to high glucose burden as the proximal trigger to unleash acute and chronic self-perpetuating loops, which include but are not limited to ROS-lipid peroxidation, hyperinflammation/disimmunity, AGE-RAGE toxicity, mitochondrial dysfunction, and nytrosilation end-products accumulation. The concerted action of these factors enforces fibroblasts, pericytes, and endothelial cells to a precocious senescence, arrest, and apoptosis. Indeed, the failure of the agonistic stimulation of the insulin axis deeply impacts on the biology of diabetics' cells. This is a seminal axis that connects the anabolic role of insulin via aminoacids transporters translocation and protein synthesis with cell survival and proliferation mechanisms, thus preventing apoptosis, autophagy (cells-self catabolism), and arrest. Fatal for the cells is silencing the agonistic stimulation of tyrosine kinase-growth factors receptors which would ensure cell cycle progression. At both experimental and clinical levels, the diabetic wound phenotype is the expression of countless molecular factors that operate through a complex biochemistry and lead to an aberrant cellular behavior. The pathway to chronification has not been fully elucidated but by all means it represents a form of cells' biological disobedience and entails the need of continuous surgical “cuttings” in order to transiently restore an acute behavior by “refreshing” the cellular environment. It is likely that the scarcity of insulin and growth factors-induced tyrosine kinase receptors downstream signaling may lie, at least in part, behind chronification. Diabetes takes away the resources that ensure wound cells perpetuation and turn-over.

Although type 2 diabetes worldwide expansion is undeniable, primary care ulcer prevention plans together with the emergence of first-line pharmaceuticals and smart devices like engineered skin equivalents will certainly prevent and reduce contemporary amputation figures.

## Abbreviations

GFs/TK-R's: Growth factors/tyrosine kinase receptors
MMPs: Matrix metalloproteases
ECM: Extracellular matrix
ROS: reactive oxygen species
OXPHOS: oxidative phosphorylation
TNF-*α*: Tumor necrosis factor
FOXO: Forkhead box O family of transcription factors
E.R. Stress: Endoplasmic reticulum stress
AGE: Advanced glycation end-products
RAGE: receptor for the AGE.
